# The Influence of Post Weld Heat Treatment Precipitation on Duplex Stainless Steels Weld Overlay towards Pitting Corrosion

**DOI:** 10.3390/ma12203285

**Published:** 2019-10-10

**Authors:** Bernard-Maxmillan Sim, Tang-Sai Hong, Mohamed Arif-Azmah Hanim, Edwin-Jong Nyon Tchan, Mahesh-Kumar Talari

**Affiliations:** 1 Faculty of Engineering, University Putra Malaysia, Serdang 43400, Malaysia; azmah@upm.edu.my; 2 Faculty of Engineering and Science, Curtin University of Malaysia, Miri 98000, Malaysia; 3 Department of Metallurgical and Materials Engineering, National Institute of Technology Warangal, Telangana 506004, India

**Keywords:** duplex stainless steel, PWHT, dilution, weld overlay, solution annealing, pitting corrosion and ferrite count

## Abstract

Duplex stainless steels (DSSs) are complex materials and they have been widely used in the marine environment and gas industries, primarily offering a better resistance of pitting corrosion and good mechanical properties. In the present work, the effects of heat treatment on duplex stainless steel (DSS) weld overlay samples that were heat treated at three different temperatures, namely 350 °C, 650 °C, and 1050 °C, and followed by air cooling and water quenching were studied. Stress relief temperature at 650 °C had induced sigma phase precipitation in between delta ferrite and austenite (δ/γ) grain boundaries, resulting in the loss of corrosion resistance in the weld metal. Interestingly, post weld heat treatment (PWHT) test samples that were reheated to solution annealing temperature had shown no weight loss. The ferrite count determination in the region of weld metal overlay increased at hydrogen relief and decreased at stress relief temperatures due to slow cooling, which is more favorable to austenite formation. The amount of ferrite in the weld metals was significantly reduced with the increment of solution anneal temperature to 1050 °C because of sufficient time for the formation of austenite and giving optimum equilibrium fraction in the welds.

## 1. Introduction

Duplex stainless steel (DSS) has adequate weldability, higher strength, and good resistance in pitting corrosion for marine environment, petrochemical refineries, and offshore oil and gas industries. However, the material behavior is dependent on its microstructure, which is strongly influenced by heat treatments, such as welding heat inputs, cooling rates, and high temperatures that may lead to influence metallurgical changes [1] and increase the susceptibility to localized corrosion dramatically, as reported [2]. This process approach will allow for corrosion resistant alloy (CRA) weld overlay to provide corrosion resistance protection on low alloy carbon steel substrate. The alternative method for improving the material’s pitting corrosion resistance with reliable and affordable costs is to use DSS CRA weld overlay. Cladding had been proven to be more cost effective, while considering their higher strength, better long term reliability, and light weights [3]. This process approach will allow for DSS CRA weld overlay to provide corrosion resistance protection on low alloy carbon steel substrate.

In offshore oil and gas fields, most of weld overlay that were cladded on carbon steel pipe materials are generally made by austenitic stainless steels and nickel alloy [4,5]. DSS CRA weld overlay had not been successfully accepted by the oil and gas operators due to some inherent problems in achieving pitting and crevice corrosion resistance caused by intermetallic precipitations [6,7]. Heat treatment was not commonly applied for stainless steel materials; local post weld heat treatment (PWHT) onto DSS materials will have significant impact for intermetallic precipitation, such as Alpha Prime, Chi phase, Sigma Phase, and secondary austenite [8,9,10]. The objectives of this research are to analyze and evaluate the behavior of pitting corrosion resistance for DSS 2209 weld overlays before and after heat treatment. ASTM G48 is a standard test method for pitting and crevice corrosion resistance of stainless steels and related alloys by use of ferric chloride solution [11] with the supplementary client’s specifications to assure stricter compliance [12,13]. Testing in ferric chloride solution allows for a quick assessment of pitting corrosion resistance of stainless steels; there are similarities between ferric chloride solution and the environment within a corrosion pit that might develop during service in chloride solutions, e.g., seawater. Hence, this test has become accepted as a quality control or ranking test for stainless alloys and welds. Further investigation on the possible root causes pitting corrosion resistance property of metallurgical bonded DSS CRA weld overlay has also been conducted by performing dilution and ferrite count, as per ASTM E562 requirements [14].

## 2. Materials and Methods

The material that was used in this research study was a low alloyed steel, DMR 249A chemical composition, as given in Table 1, with the dimensions 400 mm (L) × 400 mm (W) × 20 mm (T). Welding electrode ER2209-16 (Ø4.0 mm) is designed for joining 22% chromium duplex stainless steels, including duplex CRA weld overlay onto carbon steel substrates or other grades of stainless. It has a smooth running arc that deposits high tensile strength, with resistance to stress, corrosion, cracking, and pitting in environments containing hydrogen sulfide and chlorides [15]. Table 1 stipulated the chemical composition of material and welding consumable in shield metal arc welding (SMAW) process. The welding parameter, as shown in Table 2, was carefully studied to maintain the bonding strength of CRA weld overlay and steel substrate. The weld metal thickness was between 2 mm and 3 mm, and double layers were welded to provide the resistance of pitting corrosion. Spark optical emission spectrometer (Spectrolab, Boschstrasse, Germany) was used to analyze, measure, and quantify the chemical elements; a total of three points of spark tests were performed by using an SPECTROLAB metal analyzer.

### 2.1. Ferrite Prediction and Measurement

The Schaeffler diagram was originally developed to predict the type of microstructures in weld metals and parent materials. It could also be used to predict the microstructure dilution of two different materials, such as stainless steels welded to carbon steels [16]. It was essential to calculate and predict the microstructures, such as ferrite, austenite, maternsite, or mixed modes, which could be available in the welded joints after thermal cycle imposed to the base metal. Kchaou et al. [17] stated that the Schaeffer diagram has the ability to quantitatively predict weld microstructure while using stabilizing elements nickel equivalent, Ni_eq_ and chromium equivalent, Cr_eq_, as shown in Equations (1) and (2) are given with the weighting factor, as follows:

Ni_eq_ = %Ni + (30 × %C) + (0.5 × %Mn) (1)

Cr_eq_ = %Cr + %Mo + (1.5 × %Si) + (0.5 × %Nb) (2)

### 2.2. Process Parameter

The shield metal arc welding (SMAW) process had been utilized with direct current reverse polarity (DCRP) to provide a shallow and wider welding profile. A flat welding position was selected, as it provided higher penetration caused by gravity acceleration and significant impact to the characteristic of weld molten pool and output of geometry. The welding parameter tabulated in Table 2 was carefully studied to minimize the intermetallic formation, maintain the bonding strength, and improve the resistance of pitting corrosion. Innovative nickel rich electrodes E2209-16 were used in this research study because of the greater austenite former in the fusion zone [18].

### 2.3. Heat Treatment

Duplex stainless steel did not require preheating, ambient temperatures at approximately 25 °C to 30 °C were sufficient enough to carry out the welding works. Researchers [19,20,21] stated that the inter-pass temperature should be controlled in between 100 °C to 150 °C for standard duplex stainless steel type 2205 to achieve better resistance in pitting and crevice corrosion. Two sequences of heat treatment, as indicated in Table 3, were performed on the prepared samples. The initial heat treatment was to provide hydrogen relief condition at 350 °C and stress relief at 650 °C on the test samples to improve accumulated residual stain that was caused by thermal cyclic deformation, intermetallic phases, such as alpha prime embrittlement, sigma and chi phase was formed at this range of temperatures [8]. Upon completion of soaking time, the test samples were cooled with the stir air and water quenching methods, respectively. The subsequent heat treatment was to reheat the PWHT test samples to solution annealing temperature at 1050 °C, this is to dissolve the secondary phases and homogenize the austenite microstructures at delta ferrite [22].

### 2.4. Pitting Corrosion Resistance

The purpose of pitting corrosion test was to evaluate whether intermetallic had been formed at final manufacturing was susceptible to corrosion. ASTM G48 Method A was commonly used for the qualification of DSS, highly alloyed CRAs, and weld. Industry standards agreed that for weld procedure qualification of weld overlays the surface of the test specimen shall be representative for the weld overlay at the minimum distance from the fusion line to be qualified [23,24]. Upon completion of PWHT, the test samples were sectioned to the dimension of 30 mm × 25 mm and the low alloy carbon steel substrate was removed, as it was not part of pitting corrosion test. The bottoms of weld overlay samples were coated with surface tolerant epoxy to prevent corrosion and abrasion during the ferrite chloride test. The test samples were weighed by using a precision weight balance with capability of 1 mg accuracy; before immersing the test sample into test solution, the test specimen was measured for total area of interest before being loaded into ferric chloride solution consisted dissolve 100 g of reagent grade ferric chloride (FeCl_3_·6H_2_O) in 900 mL of Type IV reagent water [25]. The prepared test solution was filtered with glass wool or equivalent filter paper to separate the insoluble particles. The prepared test specimens were immersed into the test solution and seated on the cradle, the test temperature for duplex 2205 material was 25 ± 1 °C for 24 h, in accordance with client specifications [23]. The removed test specimens were rinsed and cleaned with water in an ultrasonic bath. To determine the weight loss, the specimens were reweighed, and the test specimens were examined and analyzed at low magnification of least 20× zooming to identify the location of pitting corrosion and other damage mechanisms.

### 2.5. Volume Fraction Determination

ASTM E562 is standard test method for determining volume fraction by systematic manual point count, this standard test method was utilized for ferrite point counting in duplex stainless steel weld metal and materials, it had been traditional used and recognized by the industries. This quantitative metallography test method was more reliable when compared with magnetic measure due to contiguous materials. It required suitable grids and a number of fields to determine the ferrite content in fusion zone; the volume fraction of the phase is statistically calculated through the microstructure point grids. The test samples were required to be polished and etched for ferrite detections based on the internationally accepted test method for assumption of ferrite volumetric on the surface measured. Proper selection of test grid and magnification enable the manual counting performs correctly. A clear plastic test grid was made of transparent sheet with equally spaced points or other suitable means that can be superimposed for viewing and measurement of the ferrite and austenite phases over the image produced by a light microscope have stipulated that the grid point counting shall be made of square matrix with equally spaced, and fine lines shall be made of 0.3 mm width at the intersection points and total number of grid points shall have 4 × 4 over the full range of the volume fractions for easy calculations [12].

The superimposed test grid of 16 points was used on the magnified image chosen area for weld metal and/or base metal microstructures. The number of point counting that relied on the ferrite phase; if a positive point lied on the test grid, it would be counted as one unit. If one of phases cannot be positively justified, it should be counted as one-half unit [21]. In order to view another field, the test samples were moved in a small amount at x and/or y axis by using fine or coarse adjustment knob and mechanical stage control. A second point of ferrite counts shall execute a new field; it should fall within the specified area of interest on the test specimen. If the new field was found to be outside the test area, it should be returned to its appropriate position. It was recommended to move the test piece by using overlap fields to avoid any bias in selecting the new fields. This process was repeated until it had achieved a minimum of 400 points [12,26].

The metallographic samples were etched by electro etching by using 40 g of reagent grade sodium hydroxide (NaOH) that was added to 100 g of distilled water at 1–3 Vdc for 15 s to create a contrast between ferrite and austenite phases and also to detect the presence of intermetallic precipitates [26]. Precaution was required to avoid excessive etch, as it could be covered or masked out the secondary phases at austenite grain boundaries and cause misleading interpretation of test results. The test samples were rinsed by running hot water, thoroughly washed with acetone solution, and air dried. The etched microstructures were evaluated with metallurgical microscope light at 400 to 500×, to reveal the intermetallic phase, austenite in light phase, and ferrite in dark phase [27]. The microstructures of the duplex stainless steel weld overlays and the heat affected zones wee embedded in resin and analyzed while using a field emission scanning electron microscopy (FESEM, Carl Zeiss, Jena, Germany) carry with electron backscatter diffraction (EBD). The chemical elements distribution for weld metal and dilution area was examined while using energy dispersive spectroscopy (EDS, Carl Zeiss, Jena, Germany) microanalysis system to analyze the active chemical elements on the individual phases (austenite-ferrite) and determine for any anomalies.

## 3. Results and Discussion

### 3.1. Pitting Corrosion Properties of Weld Metals

**Figure 1:**
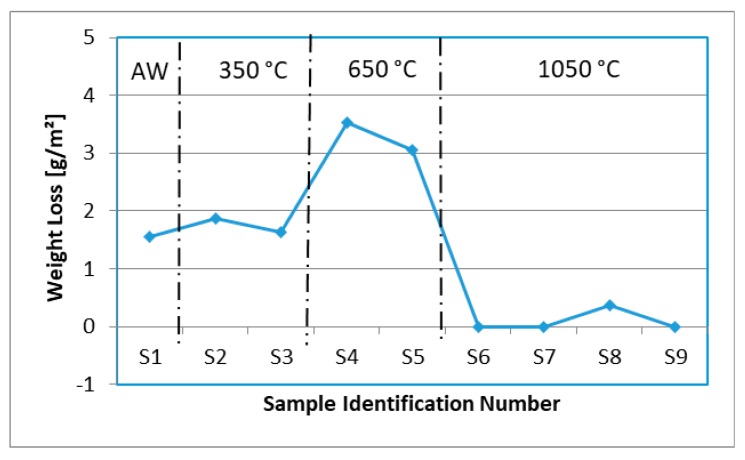
Comparison of weight loss per unit area in different heat treatments.

The pitting corrosion test was performed in accordance with ASTM G 48 Method A requirements; the test specimens were immersed into ferric chloride solution to the equilibrium temperature of 25 ± 1 °C for 24 h, in accordance with client specifications [13,23]. The initial results were obtained for calculating the area and mass loss. At the end of the 24 hours test period, all of the specimens were removed from ferric chloride solution, cleaned, rinsed, and scrubbed with a soft bristle brush under running water and dried to remove any corrosion properties. The results that are indicated in Figure 1 had displayed that sample no.1 has weakened pitting corrosion resistance by weight loss of 1.56 g/m^2^ and that pitting preferentially occurred at ferrite grain in the fusion zone. The minor weight loss was caused by a deterioration of chromium oxide; no evidence of pits was observed. PWHT was applied to improve the mechanical properties of substrate material of weld overlays. It could be observed that the weight loss per unit area g/m^2^ had gradually increased with the increment of PWHT temperatures as well as the method of cooling being applied. The sample no. 2 (350 °C AC) and no. 4 (650 °C AC) were both cooled by stirred air and indicated significant amounts weight loss at 1.87 g/m^2^ and 3.53 g/m^2^, respectively.

**Figure 2:**
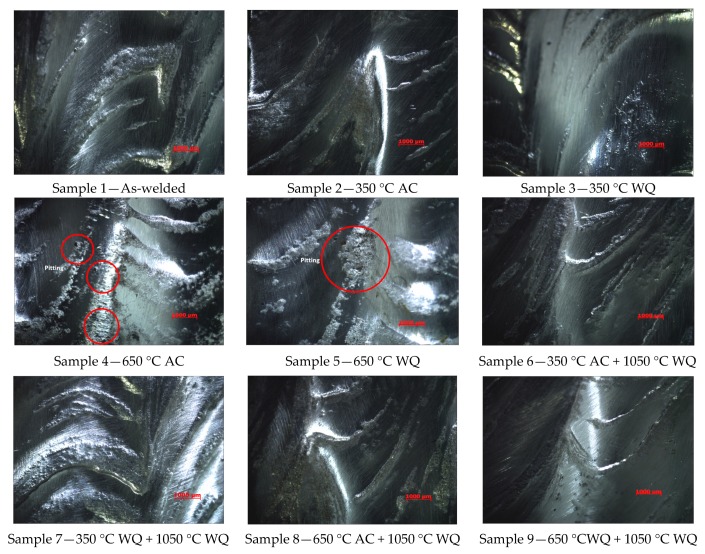
Pits morphology with optical image at 20× magnification.

Sample no. 3 and no. 5 were cooled in water quenching method in which had indicated that the weight loss per unit area was about 12.3–13.6% lower than at the air cooled condition; the actual values were obtained at 1.64 g/m^2^ and 3.05 g/m^2^. The tests results had showed that all weight losses fulfill the client standard requirements, because the metal loss per unit area has not exceeded 4 g/m^2^ [23,26]. Samples 2 to 3 weight loses are very close to sample 1 (as-welded); these were caused by deterioration of chromium oxide, as shown in Figure 2. Sample 4 and 5 could be associated with the presence of sigma phase precipitation formation in between delta ferrite and austenite (δ/γ) grain boundaries, resulting in the loss of corrosion resistance in the weldment.

**Figure 3:**
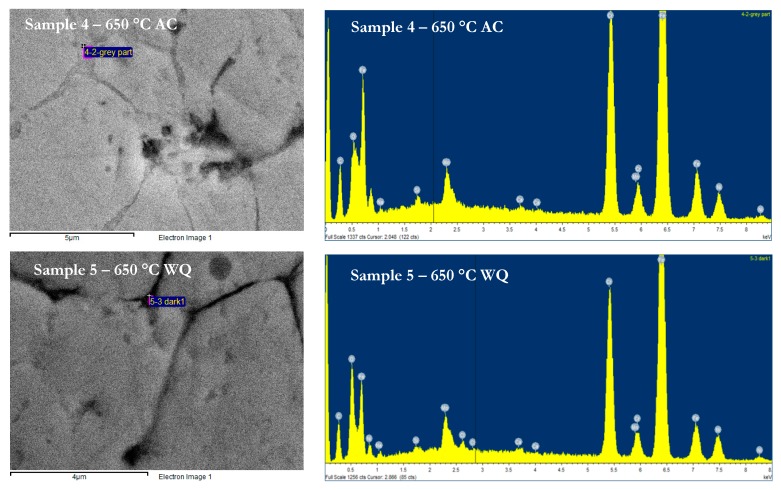
The precipitation behaviors of sigma phase in the temperature of 650 °C.

These sigma phases aggressively precipitate at the temperature range from 550 °C to 900 °C. Table 4 tabulates the ferrite and austenite (δ/γ) phase boundaries with a high intensity of chromium and molybdenum contents. Sigma phase precipitation occurred at dislocations within the ferrite grains. After nucleating from the ferrite and austenite (δ/γ) phase boundary, the sigma grows into the ferrite. Chromium starts to diffuse from the ferrite to sigma and causes the ferritic lattice to become unstable and transform into austenite. At longer times, the presence of sigma phase grain boundary could lead to intergranular corrosion when it is exposed to marine environment conditions. Therefore, it was recommended to improve the pitting corrosion resistance of weld metal with solution annealing temperature. Additionally, at the interface, the EDX full spectrum pattern had shown the two stronger peaks of Fe and Cr similar ration, as indicated in Figure 3. These parallel similar peaks had clearly illustrated the formation of intermetallic Fe-Cr compound beside the high Mo content.

**Figure 4:**
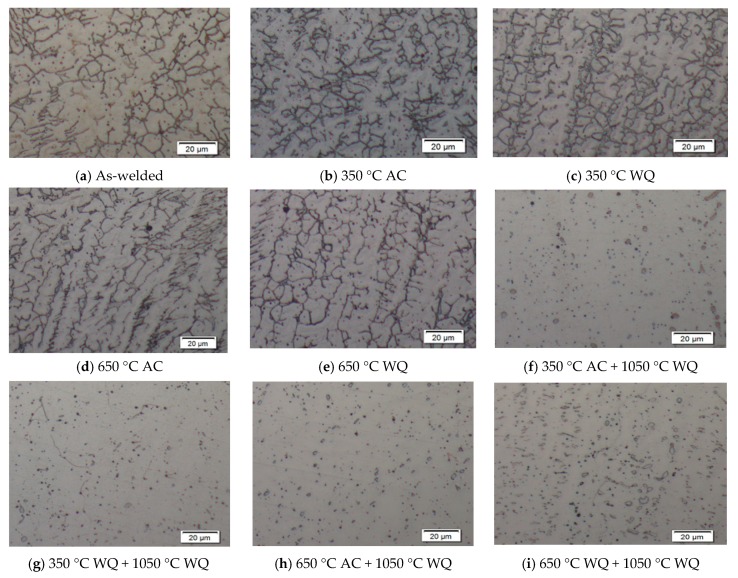
SEM micrographs of the fusion zone of various PWHT (350 °C, 650 °C and 1050 °C).

Test samples no. 6 and 7 were initiated with hydrogen relief temperature. Similarly with test sample no. 8 and 9, both were initiated with stress relief temperature, and later all of these test samples were heat treated at solid solution annealing temperature (1050 °C) for 2 h to transform ferrite to homogenize austenite microstructure and to dissolve or eliminate intermetallic phases in the weld metal. The weld overlay microstructures were being restored and exhibited better corrosion resistance, as shown in Figure 4f–i; the amount of weight loss for sample no. 6 to 9 has been significantly to zero, except for sample 8 with minimal loss at 0.36 g/m^2^ due to coating failure during handling, as highlighted earlier that the bottoms of weld overlay samples were coated with surface tolerant epoxy to prevent corrosion.

### 3.2. Volume Fraction of Ferrite

**Figure 5:**
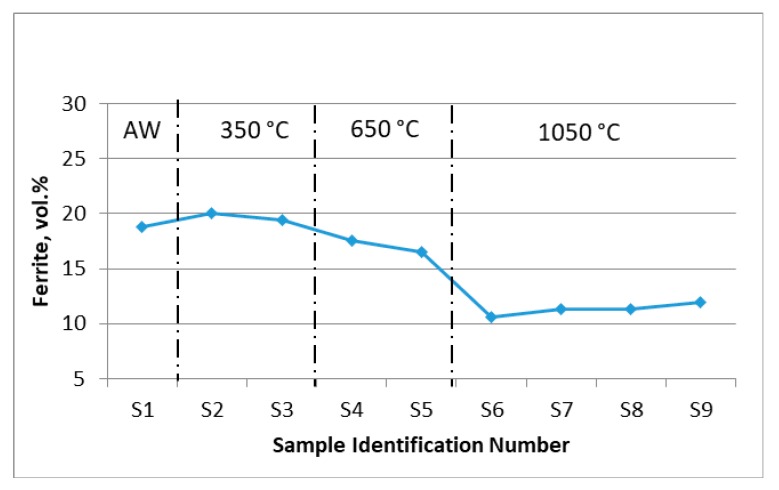
Ferrite count (vol.%) as per ASTM E562 for post weld heat treatment (PWHT) and solution heat treatment.

The effect of ferrite contents on atmospheric pitting corrosion of duplex stainless steel consumables grade 2209 was investigated with a series of various PWHT and solid solution annealing. The content of ferrites was gradually increased with incremental PWHT temperatures. Figure 5 illustrated the development of ferrite volume fraction with the effect of PWHT and solution annealing temperatures. The results had shown that the ferrite content determination in region of weld metal overlay was increased at hydrogen relief temperature and decreased at stress relief temperature. At 350 °C with the air cooled method, the ferrite counted at 20.0 ± 1.78 vol.% and water quench method, there was a slightly reduction of ferrite content at 19.4 ± 2.05 vol.%. Similarly, with stress relief temperature at 650 °C, ferrite content indicated at 17.5 ± 1.57 vol.% for air cooled and 16.3 ± 1.34 vol.% for water quenching, which is due to slow cooling that is more favorable to austenite formation. However, the test samples with solution annealing temperature at 1050 °C with water quench method had shown lower ferrite contents at 10.6 ± 1.36 vol.% to 11.9 ± 0.82 vol.%; this provided sufficient time for the formation of austenite, giving optimum equilibrium fraction in the weld and dissolve secondary phases.

Figure 4 indicates the microstructure evolution of weld metal overlays after successfully conducted with PWHT. Sample no. 2 and 3 (Figure 4b,c) showed the grain boundary austenite (GBA) and widmanstatten austenite were slightly decreased with PWHT at 350 °C as compared to the as-welded Sample no.1 (Figure 4a). Sample 4 and 5 showed that the volume fraction of ferrite were slightly deceased in weld metal, which could be due to the precipitated ferrite phase were dissolved into gamma phase. By increasing the solid solution anneal temperature at 1050 °C with 2 h of holding time, the austenite grains have not showed any favorable growth direction. Sample no. 6 to 9 (Figure 4f–i) showed that no secondary precipitation was present and a clear profile of austenite microstructures was observed.

### 3.3. Dilution of Weld Metal

**Figure 6:**
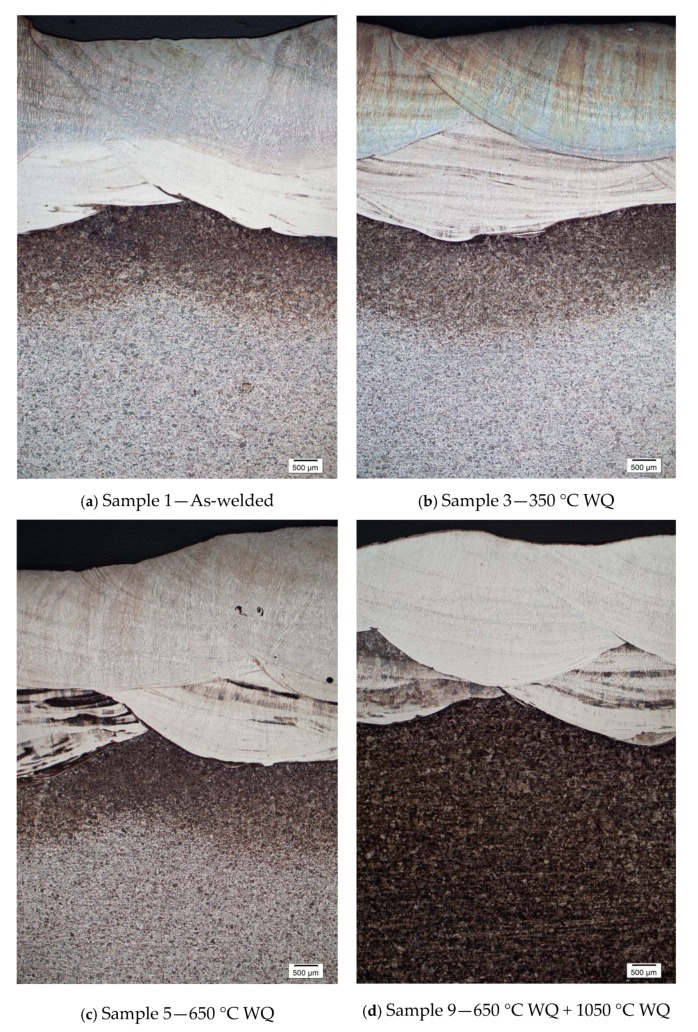
SEM image of heat affected zone (HAZ) for weld metal and their dilution area.

The composition of a duplex stainless steel weld overlay was strongly affected by the base metal and filler metal compositions, especially the dilution between the interfaces of the two materials. The dilution of weld bead chemistry had been predicted by using the 30% dilution approach, as recommended [19]. The geometrical plotting method was employed to estimate the area of dilution between the two materials; the results of the PWHT samples were obtained in the range of 30.98% and 33.14% after the welding. The dilution of duplex stainless steel weld overlay at solid solution annealing temperature at 1050 °C by water quenching has indicated the disappearance of heat affected zone, as shown in Figure 6d.

## 4. Conclusions

The pitting results showed that the as-welded sample has weakened pitting corrosion resistance by a weight loss of 1.56 g/m^2^ at the fusion zone; the minor weight lost is caused by surface deterioration of chromium oxide. The weight loss has gradually increased with the increment of heat treatment temperatures, hydrogen relief at 350 °C showed minor weight loss and stress relief at 650 °C has indicated a higher weight loss with the presence of sigma phase precipitation formation in between the delta ferrite and austenite grain boundaries. The solid solution annealing at 1050 °C with 2 h soaking time has shown that the weld metals are being restored with no significant of weight loss; the results can relate with lower ferrite contents in the weld metals. Volume fraction of ferrite in region of weld metal overlay was increased at hydrogen relief and decreased at stress relief temperatures due to slow cooling, which is more favorable to austenite formation. However, the amounts of ferrite were significantly reduced with the increment of solution anneal temperature due to sufficient time for the formation of austenite and giving optimum equilibrium fraction in the welds. The dilution of the DSS weld overlay had a slight increment in hydrogen heat treatment and a minor reduction for stress relief heat treatment. Solution annealing at high temperature, followed by water quenching, showed the disappearance of heat affected zone in micrographs.

## Figures and Tables

**Table 1:** Chemical elements of material grade DMR 249A and electrode E2209-16.

Test Materials	Chemical Composition, wt%									
	C	Cr	Ni	Mo	Mn	Si	Nb	Ti	Cu	N
Electrode (E2209-16)	0.014	23.49	10.45	3.55	1.32	0.53	0.012	0.028	0.051	0.16
Base Metal (DMR 249A)	0.110	0.30	1.05	0.05	1.65	0.40	0.05	0.06	0.30	-

**Table 2:** Welding Parameter for Duplex Stainless Steel (DSS) weld overlay.

Electrode	Current Polarity	Ampere (A)	Voltage (V)	Travel Speed (mm/min)	Heat Input (kJ/mm)
E2209-16, Ø4.0 mm	DCEP	120–125	22–25	130–145	1.22–1.29

**Table 3:** Heat treatment conditions.

Heat Treatment	Sample No.	Holding		Cooling Method (s)
		Temperature	Time	
PWHT	S1	Without heat treatment (As welded condition)		
	S2 & S6	350 °C	25 mm/h	Air Cool
	S3 & S7			Water Quench
	S4 & S8	650 °C	25 mm/h	Air Cool
	S5 & S9			Water Quench
Solution Annealing	S6	1050 °C	2 h	Water Quench
	S7			Water Quench
	S8	1050 °C	2 h	Water Quench
	S9			Water Quench

**Table 4:** Semi-Quantitative Analysis obtained from EDX Spectra.

Sample ID	PWHT	Mass Loss	Cr	Mo	Mn	Fe	Ni	Si	Type of Intermetallic Phase
	°C	g/m^2^	wt%						
4	650 AC	3.30	22.58	4.50	0.93	59.82	6.60	0.53	Sigma
5	650 WQ	3.13	21.75	5.03	0.73	53.45	7.42	0.26	Sigma
